# microRNAs in viral acute respiratory infections: immune regulation, biomarkers, therapy, and vaccines

**DOI:** 10.1186/s41544-018-0004-7

**Published:** 2019-02-14

**Authors:** Stephen A. Leon-Icaza, Mingtao Zeng, Adrian G. Rosas-Taraco

**Affiliations:** 1 Department of Immunology, Faculty of Medicine, Universidad Autónoma de Nuevo León, Monterrey, NL Mexico; 2 Center of Emphasis in Infectious Diseases, Department of Biomedical Sciences, Paul L. Foster School of Medicine, Texas Tech University Health Sciences Center El Paso, 5001 El Paso Drive, El Paso, TX 79905-2827 USA

**Keywords:** miRNA, Viral ARI, Rhinovirus, Influenza virus, hMPV, Coronavirus, RSV

## Abstract

MicroRNAs (miRNAs) are single-stranded RNAs of 17–24 nt. These molecules regulate gene expression at the post-transcriptional level and are differentially expressed in viral acute respiratory infections (ARIs), which are responsible for high morbidity and mortality around the world. In recent years, miRNAs have been studied in order to discover anti-viral ARI drug targets as well as biomarkers for diagnosis, severity, and prognosis. This review presents an analysis of the regulatory response to viral ARIs of miRNAs, including their participation in the innate immune response, their utility as biomarkers, and their potential for future therapies and vaccine development.

## Introduction

miRNAs are small molecules (17–24 nt) of non-coding, single-stranded RNA that inhibit the translation of mature messenger RNAs (mRNAs) [1]. There are more than 1881 miRNA precursor sequences in the human genome, allowing the generation of 2588 mature miRNAs according to miRBase 21.0 [2]. These molecules regulate around 60% of gene expression, they are present in a great number of biological processes, and their potential role as biomarkers in diagnosis and treatment is being explored [3]. Among the biological functions of miRNAs are maintenance of the epithelial cell barrier in the respiratory tract and regulation of anti-viral host defense [4]. Viral acute respiratory infections (ARIs) are the most common cause of acute respiratory symptoms (e.g., flu and bronchitis), and many of these infections have been linked to the exacerbation of symptoms in chronic respiratory diseases such as asthma [5].

The immune response against respiratory viruses, such as human rhinovirus (hRV), influenza virus (IV), human metapneumovirus (hMPV), human coronavirus (HcoV), and respiratory syncytial virus (RSV), is associated with altered expression of several miRNAs, and changes in the expression profile of the miRNAs in epithelial cells may contribute to the pathogenesis of acute as well as chronic respiratory diseases [4]. This review will analyze the biogenesis of miRNAs and their role in the regulation of viral ARIs.

## Biogenesis of miRNAs

The biogenesis of miRNAs involves a series of complex steps that are encoded in intergenic regions, introns, and exons of the genome [6]. First, the pri-miRNA which is nearly identical to mRNAs but lacks the translation start codon (AUG) is transcribed in the nucleus by RNA polymerase II. Folding into a characteristic stem-loop structure [7], the pri-miRNA binds to the double-stranded RNA-binding domain (dsRBD) of the protein known as the DiGeorge syndrome critical region of gene 8 (DGCR8) and is then cleaved by the ribonuclease III Drosha (also known as RNASEN), which releases an intermediate stem-loop structure of 60–70 nt known as the pre-miRNA. After cleavage, the pre-miRNA retains a phosphate at the 5′ cap and two nucleotides that protrude at the 3′ cap. Exportin-5 and RanGTP are then involved in pre-miRNA transportation to the cytoplasm [8]. Finally, the pre-miRNA in the cytoplasm is cleaved by the Dicer ribonuclease, which mainly recognizes the 5′ phosphate, generateing a double-stranded RNA (dsRNA) of approximately 22 nt (known as the mature miRNA). This molecule is then uncoiled by a helicase, producing two mature, single-stranded miRNAs that are recognized by the Argonaute protein 2 (Ago 2), which is an essential component in the RNA-induced silencing complex (RISC) [9]. The mature miRNA assembled onto RISC can bind by complementarity to the untranslated 5′ or 3′ regions of their target mRNAs, which induces the degradation or translational repression of the mRNA [10].

## The role of miRNAs in the innate anti-viral response

The host innate immune response is the first line of defense against all pathogens. A large variety of cells, such as epithelial cells [11], dendritic cells, granulocytes, monocytes, macrophages, and natural killer cells, play an important role in the immune response [12, 13]. Some miRNAs are produced during viral infection, modulating the function of all the previously mentioned cells.

**Fig. 1:**
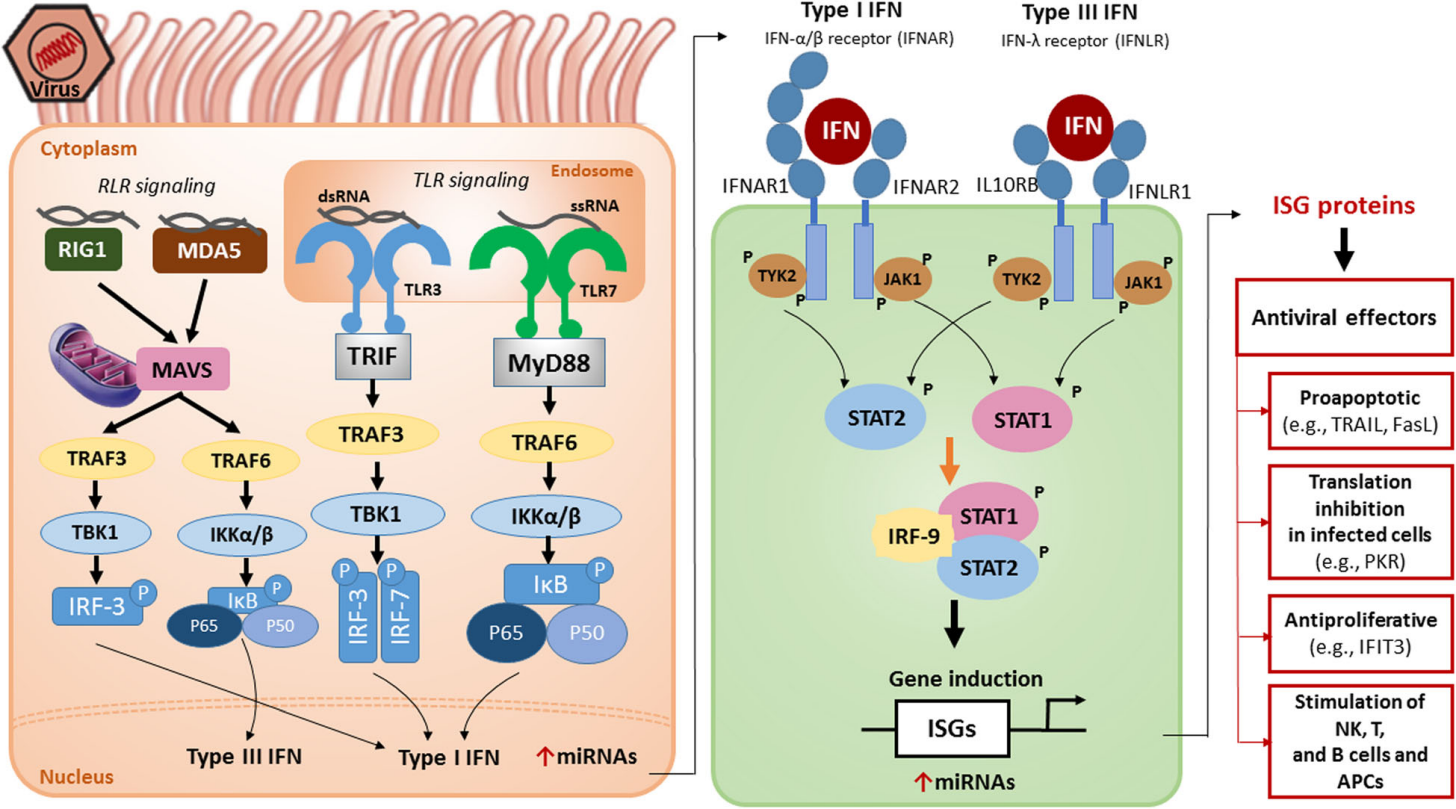
The antiviral innate immune response. Left. The NF-κB pathway. Right. The IFN pathway

Viral genetic material triggers activation of the host innate immune responses, and this material is recognized by several pattern-recognition receptors (PRRs), such as Nod-like receptors (NLRs), RIG-like receptors (RLRs), and, most importantly, Toll-like receptors (TLRs, Fig. 1) [14, 15]. The interaction of genetic material with each of these receptors depends on whether the material is DNA or RNA and whether the RNA is single stranded or double stranded [16]. It is well known that respiratory viruses mainly interact with RLRs (such as RIG-I and MDA5 at the cytoplasmic level) [17] and with TLRs (such as TLR3, TLR7, TLR8, and TLR9 at the endosomic level as well as TLR2 and TLR4 at the surface of the cell membrane) [18, 19], which culminate in two signaling pathways. The first triggers activation of the NF-κB transcription factor, which initiates the transcription of pro-inflammatory cytokines, while the second is linked to activation of interferon regulatory factors (IRFs), which promote type I and III interferon gene expression (Fig. 1) [20], and miRNAs modulate both of these pathways (Table 1).

**Table 1:** The miRNAs involved in viral acute respiratory infections (ARIs), their pathways, and the targets that they regulate [2, 83]

Virus	miRNAs	Pathways and targets
RV	miR-23b	Pathways: Cancer, Inflammation Targets: SPRY2
	miR-128	Pathways: Apoptosis, Cancer, Inflammation Targets: BMI1, BAX
	miR-155	Pathways: NFKβ, IRFs, Inflammation Mediators, Macrophage Inflammatory Response, Vascular Inflammation, Interleukins, TLRs pathways Targets: TNF, TAK1, IKKϵ, SOCS1, RIPK1, IRAK3, IKBKE, IRF8, SMAD5
RSV	let-7b	Pathways: Innate immune response, Cancer Targets: CASP3, SMAD2, SMAD4, TGFBR1, IL10, STAT3, TLR4
	let-7d	Pathways: IL6 / STAT3 Signaling Targets: CASP3, SMAD2, SMAD4, TGFBR1, IL10, STAT3, TLR4
	let-7i	Pathways: Cancer, Innate immune response Targets: CASP3, SMAD2, SMAD4, TGFBR1, IL10, STAT3, TLR4.
	miR-24	Pathways: Apoptosis, Cell proliferation Targets: E2F2, Myc, INK4a, CASP9
	miR-26b	Pathways: Inflammatory Response (IL-1), Pro-apoptotic, Cancer Targets: SMAD1, SMAD4, PTEN, TAF12
	miR-27a	Pathways: Pro-apoptotic, Cancer, Wnt signalling pathway Targets: IL10, SOCS6
	miR-27b	Pathways: Cancer, Innate immune response Targets: IL10, SOCS6, EGFR
	miR-29c	Pathways: Apoptosis, Wnt signalling pathway Targets: IL12B, CD276
	miR-30b	Pathways: Apoptosis, Inflammation, Cancer Targets: TP53
	miR-31	Pathways: Pro-apoptotic, Cancer (tumour suppressor miRNA) Targets: TIAM1, p53
	miR-34b	Pathways: Tumor suppressor, p53 pathway Targets: TP53, SIRT1
	miR-34c	Pathways: Apoptosis, Cancer, Inflammation, p53 pathway Targets: TP53, SIRT1
	miR-125a	Pathways: Apoptosis, Cancer, Inflammatory Responses Targets: MTOR, SMAD2, SMAD4, TNF, AGO2, IL6R, MYD88, TGFBR1, TP53
	miR-125b	Pathways: Apoptosis, Cancer, Regulate TNFα Targets: TNF, IL6R, AGO2, MYD88
	miR-198	Pathways: Cancer, Cell proliferation Targets: ROCK1, SHMT1
	miR-203a	Pathways: Inflammation Mediators, Apoptosis, Cancer, Targets: BCL2L2, p63, SOCS3, AP-1
	miR-429	Pathways: Cell cycle, Insulin signaling Targets: STAT4, TGFB2, TGFBR1
	miR-520a-5p	Pathways: Innate immune response Targets: RELA, TGFBR2
hMPV	let-7f	Pathways: Cancer, Innate immune response Targets: CASP3, SMAD2, SMAD4, TGFBR1, IL10, STAT3, TLR4
	miR-16	Pathways: Apoptosis, Cancer, Inflammation Targets: MAPK3, MCL1, MYB
	miR-30a	Pathways: Apoptosis, Inflammation, Cancer Targets: TP53, UGT8
	miR-192	Pathways: Apoptosis, Cancer Targets: CD67, TYMS
IV	let-7c	Pathways: IL6 / STAT3 Signaling, Apoptosis, Cancer Targets: CASP3, SMAD2, SMAD4, TGFBR1, IL10, STAT3, TLR4
	miR-17-3p	Pathways: Cancer, apoptosis Targets: CASP7, ATM, MAPK9
	miR-221	Pathways: Pro-apoptotic, Cell migration, Proliferation, Oncogenic Targets: CD117, KIT, STAT5A
	miR-323	Pathways: Apoptosis, Proliferation Targets: KIT, STAT3
	miR-491	Pathways: Apoptosis, Cell viability Targets: BCL2L1
HcoV	miR-9	Pathways: Apoptosis, Cancer, Innate immune response, NFKβ Targets: NFKB1, JAK1, JAK2, JAK3, PRDM1
	miR-98	Pathways: Anti-apoptotic, Cancer, Cytokines, Inflammation Targets: CASP3, IL10, KRAS, MYC, Ras, SMAD2, SMAD4, STAT3, TGFBR1, TLR4
	miR-214	Pathways: Apoptosis, Cancer, Autoimmune Disorders Targets: AP-2, MAPK8
	miR-223	Pathways: Interferons & Receptors, Interleukins Targets: E2F1, IGF1R

Reports have demonstrated that miR-136 interacts at the post-translational level with RIG-I to increase pro-inflammatory cytokine production [21]. miRNAs, such as let-7, may also act directly on the mRNA of a pro-inflammatory cytokine, such as IL6, to prevent its translation [22]. More recently, it has been reported that some miRNAs act on protein-coding mRNAs in signaling pathways such as IRAK1, which is a target of miR-146a (avoiding the activation of NF-κB), or IKKϵ (avoiding the activation of IRFs) [23]. Type I interferons are also targets of miRNAs [24], which may result in loss of the antiviral state (for example, miR-466i acts on IFNα, and let-7b acts on IFNβ) [25].

The IFN pathways not only favor the anti-viral state of cells but also trigger the overexpression of certain miRNAs that inhibit the NF-κB and IRF pathways. miR-155 is an example of an miRNA with these effects, as it has as targets TAK1 (inhibiting the NF-κB pathway) and IKKϵ (inhibiting the IRF pathway) [26].

## Expression of miRNAs during respiratory infections

### Rhinovirus (RV)

Rhinovirus is the main cause of upper respiratory tract infections in children and adults, and it predominantly infects the epithelial cells of the respiratory tract [27]. Rhinoviruses are single-stranded RNA viruses with icosahedral capsids and belong to the *Picornaviridae* family [28, 29]. In the viral replication step, a dsRNA is generated that is recognized by TLR3 and RIGI [30, 31].

Bioinformatic software has been useful in predicting in silico whether certain miRNAs have viral mRNAs as targets, for which the response may be in favor or against the virus [32]. The miR-128 and miR-155 miRNAs were identified as possible regulators of the innate immune response against RV-1B [33], since they have as targets the genetic material from RV. A report demonstrated that gene silencing of these miRNAs increases RV replication by ~ 50% [34].

miR-23b is involved in the immune response against RV, as it downregulates LPR5 and VLDLR transmembrane receptor expression [4]. These receptors are used by at least 12 RV types (RV1A, RV1B, RV2, RV44, RV47, RV49, RV23, RV25, RV29, RV30, RV31, and RV62) to infect cells [35].

### Respiratory syncytial virus (RSV)

RSV contains a single strand of negative polarity [36, 37] that codes for 11 proteins (NS1, NS2, N, P, M, SH, G, F, M2-1, M2-2, and L) and belongs to the *Paramyxoviridae* family [38]. It is a common human pathogen that causes symptoms similar to those found in the common cold in adults and children. It generally affects the lower respiratory tract and is the respiratory virus most frequently isolated from children hospitalized for bronchitis. Primary infection usually causes an acute illness, while subsequent infections induce episodes of obstructive bronchitis [39–41].

RSV downregulates miR-221 expression in human bronchial epithelial cell culture, while miR-30b and let-7i expression increase after 48 h of infection. Overexpression of miR-30b and let-7i was observed in normal human bronchial epithelial cell line cultures infected with an RSV that lacks NS1 and NS2 proteins, and these proteins therefore play an antagonistic role to let-7i and miR-30b, causing inhibition of the production of type I IFN. Among the miRNAs with deregulation in the levels of expression during an RSV-A2 infection are miR-27a, miR-221, miR-339-5p, miR-453, miR-574, and miR-744, and all of these were overexpressed except for the last, which was underexpressed [42].

In a case-control study, the RSV-infected patients showed low levels of expression of miR-34b, miR-34c, miR-125b, miR-29c, miR-125a, miR-429, and miR-27b compared with control; meanwhile, miR-155, miR-31, miR-203a, miR-16, and let-7d were overexpressed. Patients were divided into three groups (severe, moderate, and mild, according to the severity of the illness), and in the mild group the miR-125a and miR-429 levels were found to be downregulated [43].

Studies have determined that RSV induces miRNA expression in at least two different ways. The first, in human monocyte-derived dendritic cells (MDDCs) and human bronchial epithelial cells, the induction of let-7b and let-7i, respectively, depends on IFN-β [44]. Second, in human bronchial epithelial cells, miR-30b is induced independently of IFN but dependently on NF-κB. Finally, RSV downregulates miR-221 expression in human bronchial epithelial cells [44].

It has been demonstrated that RSV infection in A549 cells deregulates miRNA expression, including for let-7f, miR-337-3p, miR-520a-5p, miR-24, miR-26b, miR-198, and miR-595 [45]. All these miRNAs have similar targets, including cell cycle genes (*CCND1*, *DYRK2*, and *ELF4*), a chemokine gene (*CCL7*), and the suppressor of cytokine signaling 3 gene (*SOCS3*). Moreover, a G protein of RSV increases the expression of let-7f, which acts against *CCND1* and *DYRK2*, allowing cell cycle arrest in G1, favoring viral replication. The miRNA let-7 is an important key to the induction of host genes during viral infection [45].

### Human metapneumovirus (hMPV)

hMPV is an important and recently discovered member of the *Paramyxoviridae* family [46], which also includes RSV and human parainfluenza virus [47]. The genome of hMPV lacks the non-structural genes *NS1* and *NS2* and includes eight open reading frames: 3′-N-P-M-F-M2-SH-G-L-5′ [48]. Many clinical studies have shown that hMPV causes lower respiratory tract infections in pediatric patients [49–51].

It has been reported that hMPV induces changes in the miRNA expression profile (including for let-7f, miR-4552, miR-30a, miR-16, miR-374a*, and miR-192) in the epithelial cells of the respiratory tract. In A549 cells, hMPV regulated the expression of 174 miRNAs over a period of 15 h. One of the most important miRNAs overexpressed was let-7f, which has, as a possible target, the RNA polymerase of hMPV, and thus let-7f can control viral replication [52]. More studies designed to define the role of miRNAs during in vitro and in vivo hMPV infection are needed.

### Influenza virus (IV)

Influenza is caused by a single-stranded RNA virus belonging to the *Orthomyxoviridae* family [53], and there are three types of influenza virus (IV): A, B, and C. Type A (influenza A) viruses are subclassified depending on the two proteins present on their surface, hemagglutinin and neuraminidase (H and N, respectively) [54]. There are 16 different types of hemagglutinin and 9 types of neuraminidase currently known [55, 56]. The subtypes of IV with the currently highest circulation in America are influenza A (H1N1 or H3N2) and influenza B [57].

Influenza is an acute and contagious viral respiratory disease, and its characteristic manifestations are: fever, cephalea, myalgia, coryza, sore throat, and coughing. IV has a preference for the upper respiratory tract, but in severe cases it may affect the lower respiratory tract (lungs and bronchioles) [58].

The expression of miRNAs may be altered during an IV infection. miRNAs such as miR-323, miR-491, and miR-654 inhibit influenza A H1N1 replication, and these downregulate viral gene expression in infected cells [59]. An example of this mechanism is the degradation of the PB1 mRNA (involved in viral replication) of influenza A virus by host miR-323, miR-491, and miR-654 [60]. The inhibition of expression of the M1 protein of the type A IV is regulated by let-7c in A549 cells [60, 61]. Low expression of miR-17-3p and miR-221 was found in human alveolar basal epithelial cells during IV infection [62].

### Coronavirus (HcoV)

Coronaviruses are wrapped in a coat of single-stranded RNA and positive polarity [63, 64]. They have been identified as the most frequent cause of respiratory tract infections [65], ranging from the common cold to severe acute respiratory syndrome (SARS) [66].

Coronaviruses are the causal agent of the common cold, which has a low mortality rate, because the host has a perfect mechanism for resolution of the infection in most cases [67]. This mechanism is depends critically on the OC43 protein of the coronavirus nucleocapsid [68]. Cells affected by the coronavirus activate signaling cascades, resulting in an increase in NFKB1 and miR-9 expression. NFKB1 mRNA is the target of miR-9, and this leads to the loss of translation of NF-κB; however, this outcome is avoided by the action of OC43, which binds to miR-9, allowing NF-κB translation, pro-inflammatory cytokine production, and type I interferon production, which are necessary to resolve the infection [69].

SARS, caused by SARS-HcoV, is an acute infectious disease with a significant mortality rate. Common clinical features associated with SARS are pulmonary fibrosis and pulmonary insufficiency [70]. Bronchoalveolar stem cells (BASCs) are the main cells infected by SARS-HcoV [71], which induces overexpression of miR-574-5p and miR-214. Some proteins of the viral nucleocapsid downregulate miR-223 and miR-98 expression in BASCs, which controls several stages of their differentiation as well as pro-inflammatory cytokine production [72].

## New therapies focus on miRNAs and their utility in vaccines

In this review, we have discussed how miRNA expression is altered during viral ARIs, and these miRNAs are potentially useful as biomarkers and drug targets [73]. Currently, no drug exists that increases the levels of, or inhibits, any miRNAs in viral ARIs; however, there has been some progress on other diseases. The first inhibitory drug for a specific miRNA (miR-122) was created in 2010 and, as of this writing, is in phase II trials for hepatitis C treatment [74, 75]. The first synthetic miRNA, miR-34 (MRX34), was developed in 2013 for the treatment of advanced hepatocellular carcinoma [76].

In more recent research, synthetic miRNAs have been generated that are carried by liposomes and transfected into the mononuclear cells of peripheral blood. These protocols increase certain pro-inflammatory cytokines, such as TNF-α, favoring the innate immune response [77]. The most recent application of these miRNAs has been the creation of new vaccines with attenuated viruses that are loaded with an expression cassette encoding a synthetic miRNA that targets structural proteins of the virus. The PR8-amiR-93NP virus was generated by inserting an expression cassette for miR-93 between viral genes encoding non-structural proteins in an attenuated IV, and this miRNA specifically targets the nucleoproteins of the IV. This vaccine, administered intranasally, conferred immunity against several heterologous viral strains [78]. Plants also produce miRNAs that regulate virus replication. An example is MIR2911 in honeysuckle, which inhibits the expression of the PB2 and NS1 proteins of the influenza A viruses H1N1, H5N1 and H7N9 [79].

The main challenge in the development of miRNA-based therapies is the absence of an in vivo delivery method. Currently, the most common and effective method for the delivery of small RNAs (principally siRNAs) in the respiratory tract is their aerosolization with a microsprayer [80, 81]. This approach is an area of opportunity to develop miRNA delivery for possible use in these respiratory infections [82].

## Concluding remarks

miRNAs play a crucial role in the regulation of (in favor of or against) the innate immune response in viral ARIs. This regulation clearly differs according to the causal viral agent, and it is therefore important to explore the utility of miRNAs as biomarkers and for developing treatments and vaccines.

## Abbreviations

Ago 2: Argonaute protein 2
ARIs: Acute respiratory infections
BASCs: Bronchoalveolar stem cells
DGCR8: DiGeorge syndrome critical region of gene 8
dsRBD: Double-stranded RNA-binding domain
dsRNA: Double-stranded RNA
HcoV: Human coronavirus
hMPV: Human metapneumovirus
hRV: Human rinovirus
IRFs: Interferon regulatory factors
IV: Influenza virus
MDDCs: Monocyte-derived dendritic cells
miRNAs: MicroRNAs
mRNAs: Mature messenger RNAs
NLRs: Nod-like receptors
PRRs: Pattern-recognition receptors
RISC: RNA-induced silencing complex
RLRs: RIG-like receptors
RNASEN/Drosha: Ribonuclease III Drosha
RSV: Respiratory syncytial virus
SARS: Severe acute respiratory syndrome
siRNA: Small interfering RNA
SOCS3: Cytokine signaling 3 gene
